# Copper (II) and 2,2′-Bipyridine Complexation Improves Chemopreventive Effects of Naringenin against Breast Tumor Cells

**DOI:** 10.1371/journal.pone.0107058

**Published:** 2014-09-05

**Authors:** Júlio César Conceição Filho, André Lúcio Franceschini Sarria, Amanda Blanque Becceneri, Angelina Maria Fuzer, Jaqueline Raquel Batalhão, Caio Marcio Paranhos da Silva, Rose Maria Carlos, Paulo Cezar Vieira, João Batista Fernandes, Márcia Regina Cominetti

**Affiliations:** 1 Departamento de Gerontologia, Universidade Federal de São Carlos, São Carlos, SP, Brazil; 2 Departamento de Química, Universidade Federal de São Carlos, São Carlos, SP, Brazil; Wayne State University, United States of America

## Abstract

Cancer is the second leading cause of death worldwide and there is epidemiological evidence that demonstrates this tendency is emerging. Naringenin (NGEN) is a trihydroxyflavanone that shows various biological effects such as antioxidant, anticancer, anti-inflammatory, and antiviral activities. It belongs to flavanone class, which represents flavonoids with a C6-C3-C6 skeleton. Flavonoids do not exhibit sufficient activity to be used for chemotherapy, however they can be chemically modified by complexation with metals such as copper (Cu) (II) for instance, in order to be applied for adjuvant therapy. This study investigated the effects of Cu(II) and 2,2′-bipyridine complexation with naringenin on MDA-MB-231 cells. We demonstrated that naringenin complexed with Cu(II) and 2,2′-bipyridine (NGENCuB) was more efficient inhibiting colony formation, proliferation and migration of MDA-MB-231 tumor cells, than naringenin (NGEN) itself. Furthermore, we verified that NGENCuB was more effective than NGEN inhibiting pro-MMP9 activity by zymography assays. Finally, through flow cytometry, we showed that NGENCuB is more efficient than NGEN inducing apoptosis in MDA-MB-231 cells. These results were confirmed by gene expression analysis in real time PCR. We observed that NGENCuB upregulated the expression of pro-apoptotic gene caspase-9, but did not change the expression of caspase-8 or anti-apoptotic gene Bcl-2. There are only few works investigating the effects of Cu(II) complexation with naringenin on tumor cells. To the best of our knowledge, this is the first work describing the effects of Cu(II) complexation of a flavonoid on MDA-MB-231 breast tumor cells.

## Introduction

Cancer is the second leading cause of death and there are epidemiological evidences demonstrating that this tendency is emerging worldwide. Almost 1.4 million women were diagnosed with breast cancer worldwide in 2008 with approximately 459,000 deaths recorded [1]. Breast cancer is the third most frequent cancer and one of the most common malignant diseases in women worldwide. In developing countries, it is the second highest cause of death in women after cervical cancer [2]. Excluding skin cancer, breast cancer is the most common malignancy among women, accounting for nearly 1 in 3 cancers diagnosed among women in the United States [3]. The Brazilian National Cancer Institute data show that breast cancer is the leading type of cancer in women and that, over the past 30 years, mortality has increased [4]. The number of cancer-related deaths is expected to increase 45% between 2007 and 2030, influenced in part by population growth and global aging.

In order to treat breast and many other cancer types, chemotherapy is one of the most extensively studied methods. However, its efficacy and safety remain a primary concern as well as its toxicity and other side effects [5], [6]. Another reason for concern regarding this method is the development of chemotherapy resistance, which is a major obstacle to the effective treatment of many tumor types, including breast cancer [5]. Tumor cells are found to adopt multiple mechanisms to resist drugs, such as decreased uptake, and/or enhanced efflux and altered drug metabolism. Alteration in drug targets, activation of detoxification systems, enhanced DNA repair ability, and inhibition of apoptosis are also cancer cell strategies to resist against chemotherapy drugs [7].

Analysis of the tumor expression has revealed three breast cancer sub-types: progesterone receptor-positive (PG-positive), human epidermal growth factor receptor 2-positive (HER2-positive) and triple negative breast cancer (TNBC) sub-types [8]. TNBC accounts for about 15% of all breast cancer cases and is unresponsive to standard drug regimens like hormone replacement therapy as well as anti-HER-2 compounds and has a very poor prognosis [9]. Therefore, the search for new natural products that may be used as an additional alternative to chemotherapy, in an attempt to develop more effective drugs with fewer side effects, especially for TNBC, is of great interest [10].

Flavonoids, a class of polyphenols found in fruits and vegetables, have been shown to have promising chemopreventive properties against different cancer types [11]–[14]. Flavonoids are composed of several classes such as flavonols, flavonones, flavones, flavanols, iso-flavonoids, and antho-cyanidins. Naringenin, a metabolite of naringin [15], is a trihydroxyflavanone that shows various biological effects such as anticancer [16]–[20], antioxidant [21]–[24], anti-inflammatory [25], [26], and antiviral [27], [28] activities. It belongs to the flavanone class of flavonoids and has a C6-C3-C6 skeleton. The biological activities of flavonoids depend on the degree of condensation in their structures and the position and number of substitutions, such as hydroxy groups, glucosides, isoprenyl units, homodimers, and heterodimers.

The success of cisplatin and its derivatives as anticancer agents has stimulated the development of new metal-based compounds. However, tumors usually become resistant to platinum-based drugs during the clinical treatment [29]. In this way, there is a significant need for new agents with low susceptibility to common drug resistance mechanisms in order to improve response rates and potentially prolong patient's survival. Recently, interests turned on Cu(II) complexes due to their possible medical uses as antitumor agents [30]. It was already demonstrated that the coordination of Cu(II) ion with bioactive ligands can improve drug pharmaceutical activity or reduce their toxicity effects [31], [32].

The goal of this work was to investigate the effects of Cu(II) complexation to naringenin on TNBC MDA-MB-231 cells. We demonstrate that Cu(II) complexation improved the chemopreventive effects of naringenin against this cell line. NGENCuB altered MDA-MB-231 colony formation, changing their size and number and inhibited cell proliferation with 10 fold lower IC_50_ compared to NGEN itself. In addition, NGENCuB inhibited MDA-MB-231 cell migration more pronouncedly compared to NGEN. Moreover, NGEN and more effectively NGENCuB showed an effect inhibiting the activity of pro-MMP9. Finally, we demonstrated that NGENCuB had more important effects, compared with NGEN itself, on the induction of apoptosis in MDA-MB-231 cell line, as demonstrated by DAPI coloration, flow cytometry analysis and by expression of pro- and anti-apoptotic markers by real time PCR.

## Experimental Procedures

### 1. Synthesis of Cu(II)-Bipyridine-Naringenin complex and its stability

**Figure 1:**
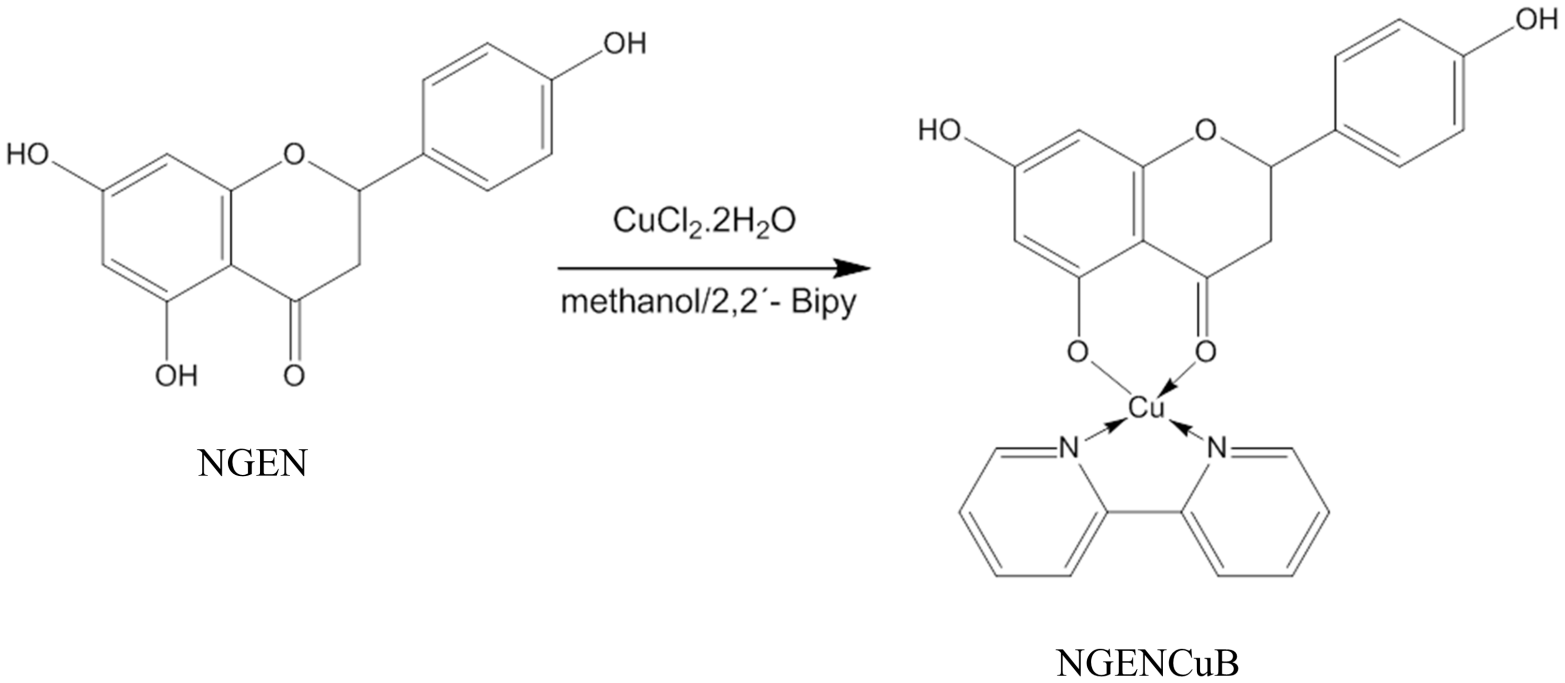
Complexation of Naringenin (NGEN) with copper (II) and 2,2′-bipyridine (NGENCuB).

(±)-Naringenin (NGEN) was obtained from Sigma-Aldrich [Product N5893, (±)-2,3-Dihydro-5,7-dihydroxy-2-(4-hydroxyphenyl)-4H-1-benzopyran-4-one or (±)-4′, 5, 7-Trihydroxyflavanone; St. Louis, MO, USA] as well as 2,2'-bipyridine (Product D216305, 2,2'-bipyridyl) and copper (II) chloride dihydrate [Product 307483, Cu(Cl_2_).2H_2_O]. Complexation of naringenin with copper and 2,2′-bipyridine as ancillary ligand (NGENCuB) (Figure 1) consisted in the preparation of an aqueous solution of copper chloride (II) [Cu(Cl_2_).2H_2_O] 1.25×10^−4^ mol (0.0213 g) in 2 mL of distilled/purified water and dropped slowly in a methanol solution of mixture of naringenin 1.25×10^−4^ mol and 2,2'-bipyridine 1.25×10^−4^ mol. The mixture was stirred for 6 hour at room temperature. The light green precipitate formed was filtered in a vacuum system, washed with acetone and dried under vacuum pump.

The Cu(II) complex with naringenin and 2,2′-bipyridine as ancillary ligand was obtained in good yield (79,0%) and was characterized by its UV-vis (intense absorption at 300 nm, ε = 2.5×10^5^ L mol^−1^cm^−1^, broad absorption at 380 nm, ε = 2.5×10^5^ L mol^−1^cm^−1^ in methanol (Agilent 8453 UV-visible spectrophotometer); IR 1602 cm^−1^ (C = O), 777 cm^−1^ (C = N), 418 cm^−1^ (Cu-O), 304 cm^−1^ (Cu-N), [BOMEM, BM-Séries Aridzone); HRMS *m/z* (%) 490.05556/492.0589 (calc. 490.0590/492.0572 [M-Cl]^+^ (26/12), 253.9665 [M-Naringenin]^+^ (100), 247.0038 [bipyridineCuCO]^+^ (29), 218.9920 [bipyridineCu]^+^ (2), 156.0679 [bipyridine]^+^. (23), (Direct infusion using solution in methanol and formic acid 0.1%, Bruker Daltonics, Billerica, EUA; modelo UltrOTOFQ - ESI-TOF); anal. calcd for CuC_25_H_19_N_2_O_5_Cl: 56.12% (C), 3.80% (H), 5.03% (N), found: CHN 57.68% (C), 3.97% (H), 4.90% (N), Fisons, model EA1108 CHNSO; TGA curves (in supplementary material; NETESCH-GERATEBAU Gmbh model TG 209 TARSUS F3) of NGENCuB shows the presence of the ligands NGEN and bipyridine on the coordination sphere of metal center and suggesting the purity of complex.

Its purity was determined by capillary electrophoresis (>99%). The experiment was carried on a Beckman P/ACE MDQ capillary electrophoresis system (Beckman Instrument Fullerton, CA, USA) equipped with a diode array detector set to 273 nm. Sample was positioned at the inlet tray, and hydro dynamically injected at 0.5 psi (3447 Pa) for 5 s. Separation was performed with 20 kV under normal polarity. Data acquisition and manipulation were performed with software provided by the manufacturer (32 Karat Gold). Uncoated fused-silica capillaries with 50 µm i.d., 375 µm o.d. and 50 cm long (43 cm from inlet to detector window; 7 cm from outlet to detector window) were used, with capillary temperature kept at 25°C by an external coating with a liquid coolant. New capillaries were rinsed with NaOH 1 M (30 min), followed by NaOH 0.1 M (30 min), MeOH (15 min) and equilibrated with buffer (20 min). First rinse of the day consisted of NaOH 1 M (10 min) followed by NaOH 0.1 M (5 min), MeOH (5 min) and equilibrated with buffer (10 min). Between runs the capillary were rinsed with NaOH 0.1 M (3 min), MeOH (4 min) and buffer (6 min). All rinses were performed with hydrodynamic injection at 40 psi. NGENBCu was solubilized in buffer sodium tetraborane 20 mM in MeOH and 50 mM SDS (sodium dodecyl sulfate)]. Its purity was determined by capillary electrophoresis (>99%) and the resulting compound had a light green solid aspect with melting point of 330±2.0°C.

The chemical stability of the complex in methanol and in DMEM medium was investigated by UV-vis. Solutions of the complex at 1, 10 and 100 µM were prepared, and the UV-vis absorption spectrum was taken at appropriate time intervals. The absorption spectrum remained unchanged at least three days.

### 2. Cell line and culture

MDA-MB-231 human breast tumor, obtained from ATCC (Manassas, VA, USA), was maintained at 37°C in 5% CO_2_ in DMEM medium containing fetal bovine serum (FBS) 10%, (Vitrocell, Campinas, SP, Brazil). MCF-10A, which are non-tumor breast cells, obtained of Rio de Janeiro Cell Bank (Rio de Janeiro, RJ, Brazil) were cultivated in DMEM/F12 medium containing horse serum (HS) 5%, EGF (0.02 mg/ml); hydrocortizone (0.05 mg/ml); cholera toxin 0.001 mg/ml); Insulin (0.01 mg/ml). Both media contained penicillin (100 UI/ml), streptomycin (100 mg/ml) and L-glutamine (2 mM).

### 3. Cell morphology

Exponentially MDA-MB-231 and MCF-10A growing cells were harvested, counted and seeded (0.6×10^6^ cells/plate) into 3 cm Petri dishes (Corning, Union City, CA, USA). Cells were allowed to grow at 37°C in 5% CO_2_ overnight and then, treated or not (control) with 1, 10 and 100 µM of naringenin (NGEN) or Cu(II) complexed naringenin (NGENCuB) for 24, 48 and 72 hours. Cell morphology was examined under an inverted microscope with amplification of 40×.

### 4. Colony formation

Exponentially MDA-MB-231 growing cells were harvested, counted and seeded (300 cells) into Petri dishes. Cells were allowed to grow at 37°C in 5% CO_2_ overnight and then, treated with different concentrations of NGEN or NGENCuB for 2 days. After this time, the medium was changed to a fresh medium without any compound. After incubation for additional 10 days the cells were rinsed with PBS, fixed with methanol for 15 minutes and stained with 0.5% crystal violet for 15 minutes. Relative survival was calculated from the number of single cells that formed colonies of >50 cells on the tenth day. The plating efficiency (PE) was calculated according to Franken et al. [33].
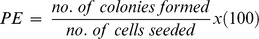



### 5. Cell proliferation

Proliferation assay was performed as described earlier [34]. Briefly, both cells lines were prepared in a concentration of 5×10^4^ cells/200 µl, in a complete medium (with 10% FBS), and plated on sterile 96 well plates for 5 hours. The culture medium was removed from the wells and a new one, supplemented with 10% FBS and containing different concentrations of NGEN or NGENCuB, was added to the wells. Cells were incubated for 48 hours under the same conditions as described above. Cell proliferation assay was performed in comparison to the wells where the vehicle compound (2.5% DMSO) was added instead of the tested compounds. After incubation, the culture medium of each well was removed and a solution containing MTT (0.5 mg/ml) was added (100 µl/well). The plates were then kept at 37°C for 4 hours and the formed crystals were dissolved in isopropyl alcohol. The absorbance was read on an ELISA plate reader at a wavelength of 595 nm. Doxorubicin was used as a positive control for cell proliferation inhibition [35].

### 6. Wound healing

MDA-MB-231 cells (1×10^5^/ml) were plated in 24-wells plates and incubated properly until the culture reached 100% of confluence. Afterwards, a straight scratch was made with a sterile pipette tip and cells were washed with culture medium to remove unbound cells and debris. Cells were incubated with NGEN or NGENCuB (1, 10 and 100 µM) for 24 and 48 hours. The cells were viewed using an inverted microscope (Lake Success, NY, USA) at 40× total magnification and captured using a Sony model XCST50 camera (Park Ridge, NJ, USA) at 0 h, 6, 12, 24 and 48 hours. Closure area of migrating cells was measured using Image J software, and it was calculated the percentage of wound closure, comparing time zero and 48 hours later, using a formula from Yue et al. [36], showing the difference between 0 h and 24 hours:
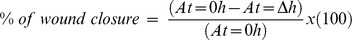



### 7. Migration

Cell migration was assessed in 24 well Boyden chambers (BD Biosciences, Franklin Lakes, NJ, USA) as described earlier [37]. MDA-MB-231 (5×10^4^) cells incubated or not with NGEN or NGENCuB (1, 10 and 100 µM) were seeded on the upper chamber in a DMEM incomplete medium and allowed to migrate for 22 hours at 37°C and 5% CO_2_ in a humidified environment. Then, the cells that remained in the upper chamber were removed using a cotton swab. The cells that migrated to the other side of the upper chamber membrane were fixed with methanol and stained with 1% toluidine blue. Cells were counted using Image J software (public domain software) in 5 fields (100× magnification) per well that essentially covered 80% of the well surface. The average number of cells from each of the triplicates represented the average number of cells that migrated in the different groups. Each experiment had triplicate wells for every treatment group and the assays were repeated three times. The mean of all results from controls was considered as 100% of migration.

### 8. Zymography

MDA-MB-231 cells (1×10^5^/well) were seeded into 12 well culture plates and cultured in a medium containing FBS 10% to near confluence (80%) of the cell monolayer. The monolayers were carefully wounded using a yellow pipette tip, and any cellular debris present was removed by washing with PBS. The wounded monolayers were then incubated in serum-free medium containing 0, 1, 10 and 100 µM of NGEN or NGENCuB for 24 hours. After this time, supernatants of the wound healing assay were collected and tested for MMP secretion as previously described [38]. Briefly, equal amounts of total protein (10 µg/lane) were subjected to electrophoresis. Zymography gels consisted of 10% polyacrylamide impregnated with gelatin at a final concentration of 1% in the presence of sodium dodecyl sulfate (SDS) under non-reducing conditions. After 2 hours of electrophoresis (80 V), the gels were washed twice for 20 minutes in a 2.5% Triton X-100 solution, and incubated at 37°C for 20 hours in a substrate buffer (50 mM Tris–HCl, pH 8.5, 5 mM CaCl_2_ and 0.02% NaN_3_). The gels were then stained with coomassie brilliant blue R-250 for 30 minutes and destained in methanol and acetic acid for 20 minutes. Gelatin-degrading enzymes were visualized as clear white bands against a blue background, indicating proteolysis of the substrate protein. The molecular mass of gelatinolytic activities was determined by comparison to reference protein molecular mass marker PageRuler Prestained Protein Ladder (Thermo Fisher Scientific, Pittsburgh PA, USA). Activity bands were identified following the previous description according to their molecular weights.

### 9. Apoptosis

#### 9.1 DAPI staining

MDA-MB-231 cells (0.1×10^6^/plate) were seeded in 12-wells plates on a 15 mm-coverslips and maintained at 37°C in a humidified incubator with 5% CO_2_ for 24 hours. In the next day, cells were treated with 1, 10 and 100 µM of NGEN and NGENCuB and incubated for an additional 24 hours period. Next, cells were washed with PBS, fixed with methanol and stained with 300 nM DAPI (Life Technologies, Carlsbad, CA) in DMEM medium without FBS for 10 minutes. Slides were mounted and fluorescence was captured in a fluorescence microscope.

#### 9.2 Flow cytometry

The apoptotic activity of NGEN and NGENCuB on MDA-MB-231 cells was analyzed by flow cytometry with the PE-Annexin V Apoptosis Detection Kit (BD Biosciences). Cells (1×10^5^) were seeded in 12 well plates in a complete DMEM medium and incubated for 24 hours. After this period the medium was removed and cells were incubated or not with different concentrations of NGEN or NGENCuB, overnight at 37°C and 5% CO_2_. MDA-MB-231 cells were harvested by trypsinization, centrifuged, washed twice with cold PBS and resuspended in binding buffer (100 µl). Cells were incubated with PE-Annexin V (5 µl) and 7ADD (5 µl) for 15 minutes in the dark at room temperature, then 400 µl of binding buffer were added to the cells which were analyzed in Accuri C6 flow cytometer (BD Biosciences). The fluorescence was quantified by CellQuest software (BD Biosciences).

#### 9.3 Quantitative Real Time PCR

MDA-MB-231 cells (1×10^6^/plate) were incubated for 24 h with or without NGEN or NGENCuB (1 µM) in Petri dishes (6 cm) at 37°C in a humidified incubator with 5% CO_2_. Total RNA was extracted using Trizol reagent (Invitrogen, Carlsbad, CA, USA). cDNAs were synthesized using the kit Enhanced Avian RT First Strand Synthesis (Sigma-Aldrich, St. Louis, MO, USA). A Rotor-Gene 6000 real-time rotary analyzer (Corbett Life Science, Australia) was used to amplify both target and internal control templates (1 cycle at 95°C for 5 min and 40 amplification cycles at 95°C for 30 sec, 55°C for 30 sec and 72°C for 45 sec). In brief, 1 µL of reverse transcribed product template, 5 µL of SYBR® Green JumpStart Taq ReadyMix (Sigma-Aldrich, St. Louis, MO, USA) and the gene-specific primer pairs at a final concentration of 500 nM for each primer, made 10 µL of reaction system. Primers used in the assay were: caspase-8 (Forward: 5′GGA TGA GGC TGA CTT TCT G3′; Reverse: 5′CTG GCA AAG TGA CTG GAT G3′), caspase-9 (Forward: 5′GCC TCA ATG CCA GTA ACG3′; Reverse: 5′GTT GTC AGG CGA GGA AAG3′) e Bcl-2 (Forward: 5′CGG GAT TCA CAG AGT ATT TG3′; Reverse: 5′GGC TGG GCA CAT TTA CTG3′). For each gene, all samples were amplified simultaneously in duplicate in one assay run. Data represent three assays in duplicate and were normalized using the comparative cycle threshold (Ct) method. A blank with water, primers and SYBR Green instead of template sample was performed. The target gene expression was normalized to GAPDH gene, as previously described [39].

### 10. Statistical analysis

Each experiment was repeated three times in triplicate and a standard error mean was calculated. Shapiro-Wilk's test was used to verify data normality. As normal distribution was present, the results were compared statistically with ANOVA. Since the ANOVA tests showed significant differences (acceptable *P* level <0.05), Tukey's or Bonferroni's significant difference post hoc analyses were performed to determine differences between simple and grouped main-effect means, respectively. The data were analyzed using Statistica software (version 8.0; Stat Soft Inc) and IC_50_ calculations were achieved through Hill's equation in the GraphPad software (version 6; GraphPad Software).

## Results

The (±)-Naringenin (NGEN), bipyridine, CuCl_2_ complex (NGENCuB) was synthesized in good yield (79.0%), its structure was characterized by FT-IR (Figure S1), HRMS (Figure S2), UV (Figure S3), TGA (Figures S4 and S5) measurements and its purity was >99% as described in the experimental (section 2.1). The stability of NGENCuB complex (Figure S4) and naringenin without complexation (Figure S5) in the dark at room temperature and in the concentrations of 1 to 100 µM was proved by the absence of changes in the electronic absorption spectra in methanol and in DMEM medium over a period of 24 hours. This is relevant in terms of developing metallodrugs in physiologic medium.

**Figure 2:**
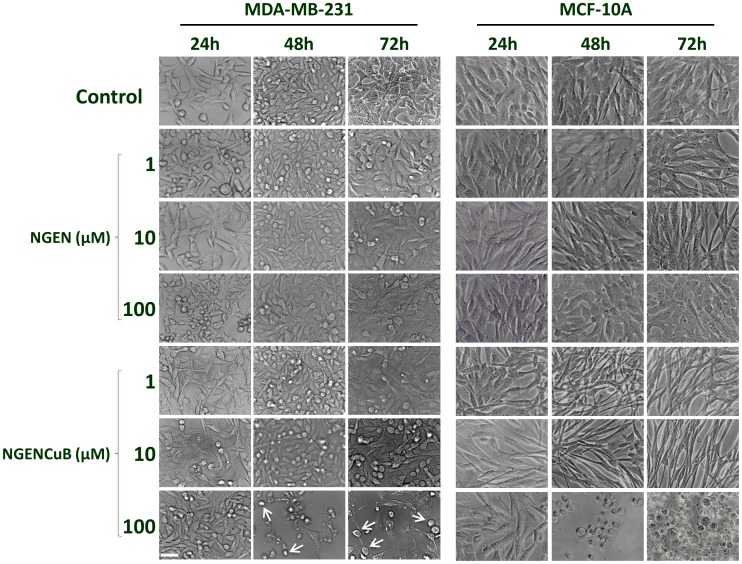
Cellular Morphology of MDA-MB-231 (right panels) and MCF-10A (left panels) control cells, naringenin (NGEN)-treated or naringenin complexed with copper (II) and 2,2′-bipyridine (NGENCuB)-treated cells. Cells were allowed to grow in a humidified incubator at 37°C in 5% CO_2_ overnight and then treated with 1, 10 and 100 µM of NGEN or NGENCuB for 24, 48 and 72 hours. Cell morphology was examined under an inverted microscope with amplification of 100×. White arrows indicate detaching round cells. Scale bar  = 100 µm.

Naringenin (NGEN) at concentrations of 1, 10 and 100 µM, did not alter MDA-MB-231 cell morphology, even after 72 hours of incubation, compared with control cells, without treatment (figure 2, left upper panels). On the other hand, naringenin complexed with Cu(II) and 2,2′-bipyridine (NGENCuB) incubated with MDA-MB-231 cells at a concentration of 100 µM for 48 or 72 hours, profoundly altered the morphology, promoting the appearing of round cells (figure 2, left lower panels, white arrows), which is an indicative of cell detachment and probably cell death. In addition, incubation of MDA-MB-231 cells with 100 µM NGENCuB for 48 and 72 hours clearly reduced cell number, compared to the control cells. We also verified the effects of both compounds on the morphology of a non-tumor cell line, MCF-10A. The results were similar of those achieved in the tumor cell line. These results demonstrate that NGENCuB is more effective altering MDA-MB-231 cell morphology and number, compared with NGEN.

**Figure 3:**
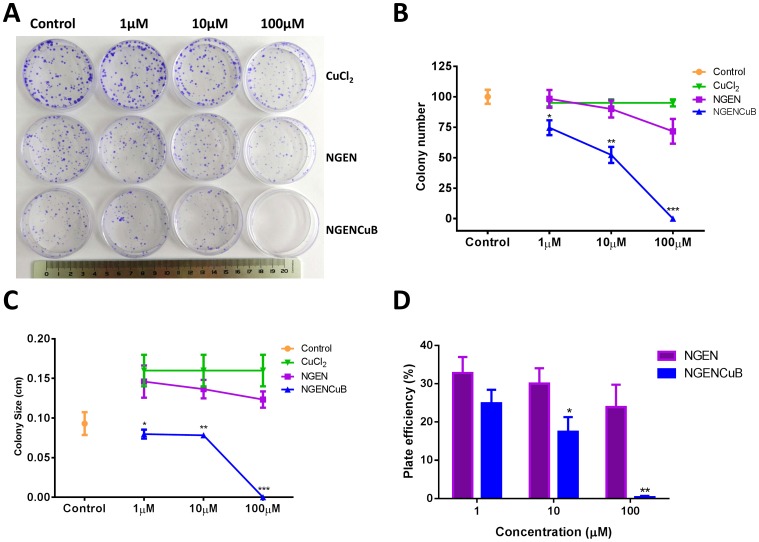
Effects naringenin (NGEN) or naringenin complexed with copper (II) and 2,2′-bipyridine (NGENCuB) on MDA-MB-231 colony formation. **A**. Clonogenic assay of untreated MDA-MB-231 cells (control) or treated with NGEN or NGENCuB. A photograph of Petri-dishes in a representative experiment is shown. **B**. Quantification of colony number. **C**. Quantification of colony size. **D**. Plate efficiency. Quantification of colony number and size was performed using Image J public domain software. Data represent means ± SD from three different experiments in triplicate. The results were compared using ANOVA, followed by a Tukey's post-hoc analysis. Asterisks represent *p≤0.05, **p≤0.01, ***p≤0.001 compared to control.

We next tested the effects of NGEN and NGENCuB for their ability to inhibit colony formation in MDA-MB-231 tumor cells (figure 3AD). The applicability of the tumor colony-forming assay for drug screening has long been accepted to investigate new antitumor drugs [40]. NGENCuB (1, 10 and 100 µM) was significantly more effective inhibiting both colony number (figure 3A, B) and size (figure 3A, C) in MDA-MB-231 cells compared with NGEN itself or untreated control cells. The plate efficiency (PE) was calculated for NGEN and NGENCuB (figure 3D) and results show that NGENCuB significantly reduced PE at concentrations of 10 and 100 µM, when compared to PE of NGEN (figure 3D). To demonstrate that copper and 2,2′-bipyridine do not have an effect inhibiting colony formation, MDA-MB-231 cells were treated with a CuCl_2_ and 2,2′-bipyridine solutions used in the synthesis of complexes at the same concentrations used for NGEN or NGENCuB (1, 10 and 100 µM) treatments. Results demonstrate that CuCl_2_ had no effect on breast tumor cells colony formation, neither in number, nor in size. However, 2,2′-bipyridine had effects only in 100 µM inhibiting the number and size of colonies (data not shown).

**Figure 4:**
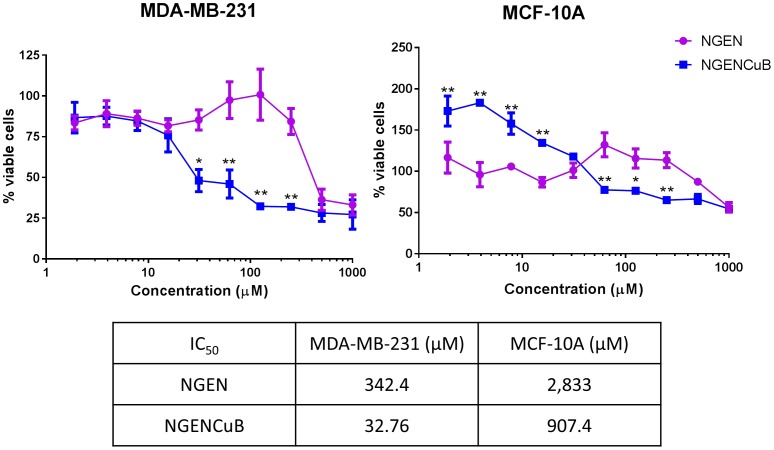
Effects naringenin (NGEN) or naringenin complexed with copper (II) and 2,2′-bipyridine (NGENCuB) on MDA-MB-231 (left) and MCF-10A (right) cell proliferation and IC_50_ values for each compound and cell line. Cells were plated in a 96-well plate and incubated with different concentrations (1 to 1000 µM) of NGEN or NGENCuB for 48 hours. Viable cells were estimated by MTT assay. Results are expressed as percent viability relative to control (untreated) cells. Data represent mean ± SD of three independent assays in triplicate. Results were compared using ANOVA, followed by a Tukey's post-hoc analysis. Asterisks represent *p≤0.05, **p≤0.01, compared to NGEN.

Next, we investigate the effects of different concentrations of NGEN and NGENCuB on MDA-MB-231 and MCF-10A cell proliferation estimated by MTT assay (figure 4). It is known that unregulated cell proliferation is one of the hallmarks of cancer as an early step in tumorigenesis. For MDA-MB-231 cell line, NGEN at concentrations of 500 and 1000 µM inhibited approximately 60% of MDA-MB-231 cell proliferation, compared with the proliferation of control cells, which was considered as 100%. In the same concentrations however, NGENCuB inhibited about 70% of tumor cell proliferation. However, NGENCuB at concentration of 30 µM (figure 4) had already an effect inhibiting approximately 50% of MDA-MB-231 cell proliferation, which was significantly different compared to NGEN-treated cells. Treatment of MDA-MB-231 cells with 65, 125 and 250 µM of NGENCuB reduced their proliferation in approximately 65 and 75%, respectively, compared to control cells (figure 4). We also tested the effects of CuCl_2_ and 2,2′-bipyridine on breast tumor cell proliferation and the calculated IC_50_ for MDA-MB-231 cell proliferation inhibition were 655.5 and 169.2 µM, respectively (results not shown).

For MCF-10A, a non-tumor cell line, NGEN at concentration of 500 µM inhibited only approximately 10% of viability, while NGENCuB at the same concentration inhibited 30% of cell viability compared to control untreated cells (figure 4). It is possible to observe that at low concentrations (2 to 32 µM) both NGEN and NGENCuB did not inhibit MCF-10A cell viability. Even in higher concentrations NGEN (60–250 µM) did not inhibit MCF-10A cell viability.

These results indicate that Cu(II) and 2,2′-bipyridine had low effects inhibiting MDA-MB-231 cell proliferation, however complexation of naringenin with CuCl_2_ and 2,2′-bipyridine improved the effects of NGEN by approximately ten times fold since the calculated IC_50_ were 342.4 µM and 32.76 µM for NGEN and NGENCuB, respectively. For MCF-10A cells the calculated IC_50_ were 2,833 µM and 907.4 µM, respectively, indicating that both compounds are more specific inhibiting tumor cell viability compared to non-tumor cells.

**Figure 5:**
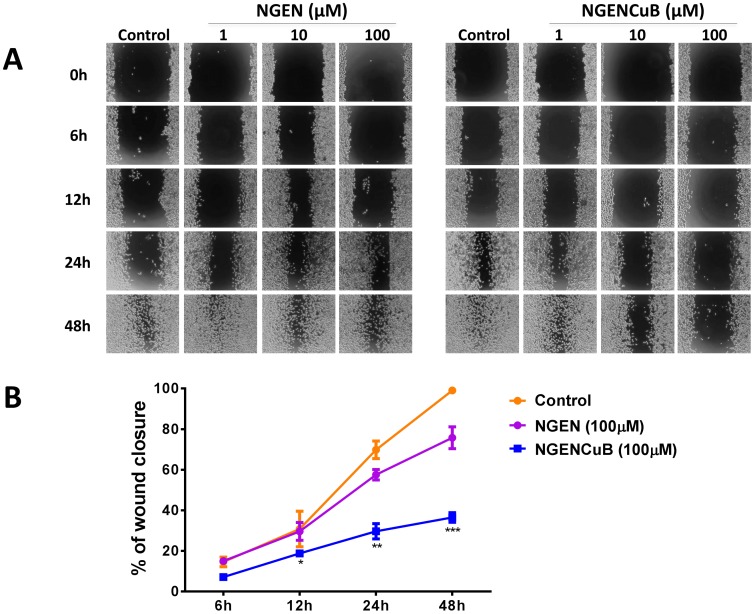
Effects of naringenin (NGEN) or naringenin complexed with copper (II) and 2,2′-bipyridine (NGENCuB) on MDA-MB-231 cell migration. **A**. Cells were scratched using a pipette tip to make gaps between cells before NGEN and NGENCuB treatment. At 0, 6, 12, 24 and 48 hours of treatment, the plates were photographed under a light microscope (40 x). **B**. Percentage of wound closure of control cells or cells treated with 100 µM of NGEN or NGENCuB. Results are expressed as percent of wound closure relative to control (untreated) cells. Data represent mean ± SD of three independent assays in triplicate. Results were compared using ANOVA, followed by a Tukey's post-hoc analysis. Asterisks represent *p≤0.05, **p≤0.01, ***p≤0.001 compared to control.

**Figure 6:**
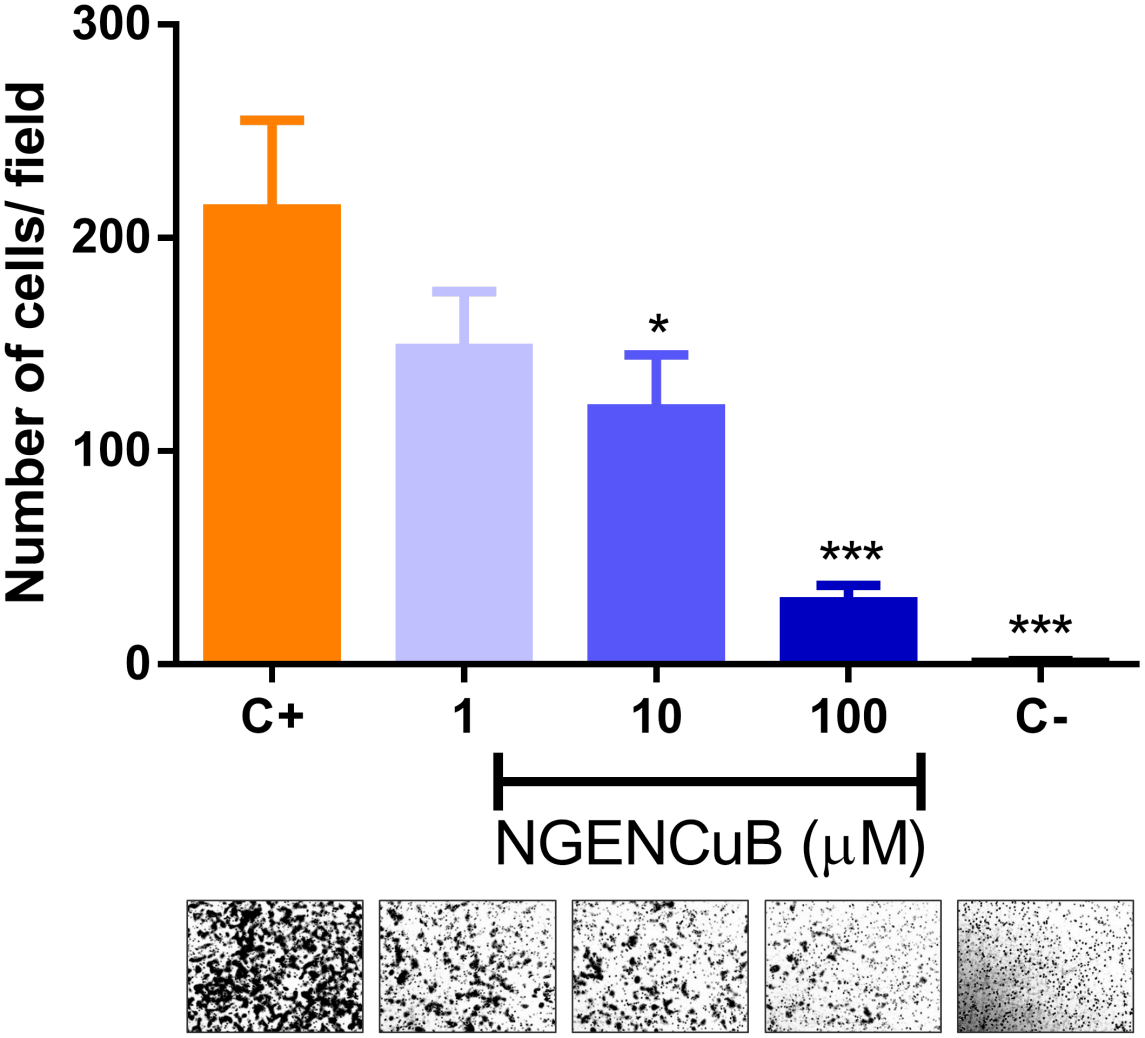
Effects of naringenin (NGEN) or naringenin complexed with copper (II) and 2,2′-bipyridine (NGENCuB) on MDA-MB-231 cell migration in Boyden chambers. Control cells or compounds-treated cells were allowed to migrate towards lower insert chambers containing FBS as chemoattractant, for 22 hours. The morphology of cells in the different treatments migrating toward FBS is in the bottom panels. Positive control (C+) represents migrating cells without any treatment, negative control (C-) represents untreated cells migrating toward an FBS-free medium. Data represent mean ± SD of three independent assays in triplicate. The results were compared using ANOVA, followed by a Tukey's post-hoc analysis. Asterisks represent *p≤0.05, **p≤0.001 compared to control (C+).

The ability to migrate and invade circulation and tissues is a key characteristic of metastasis. Therefore, we further investigated the effects of NGEN and NGENCuB on MDA-MB-231 tumor cell migration using both wound healing and Boyden chambers migration assays (figures 5 and 6, respectively). Whereas 6 hours incubation with 100 µM NGENCuB had no effect on MDA-MB-231 cell migration (figure 5A, upper panels), incubation for 12, 24 or 48 hours resulted in a clear inhibition of cell migration, compared with the migration of control cells or cells treated with the same concentration of NGEN (figure 5A, lower panels). The effects of 100 µM NGEN and NGENCuB on MDA-MB-231 cell migration to repopulate the wound are presented on figure 5B. NGENCuB at 100 µM markedly decreased the wound closure of MDA-MB-231 cells compared with control or NGEN-treated cells, with significant effects starting after 12 hours treatment (figure 5B). While control cells completely repopulated the wound after 48 hours, NGENCuB-treated cells (100 µM) only covered about 30% of the wounded area, at the same time (Figure 5B).

NGENCuB effects on MDA-MB-231 cell migration were also assessed using Boyden chambers (figure 6). In this assay, cells were incubated with different concentrations of NGENCuB, seeded on the upper chamber and allowed to migrate for 22 hours toward a lower chamber filled with complete medium. Results show that NGENCuB (10 and 100 µM) was able to inhibit MDA-MB-231 cell migration also in this model of migration assay (figure 6).

**Figure 7:**
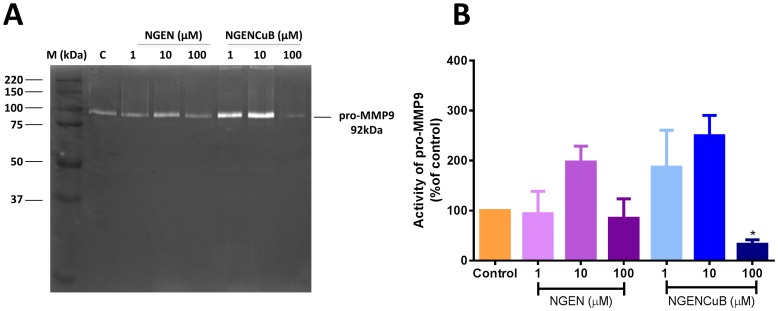
Effects of naringenin (NGEN) or naringenin complexed with copper (II) and 2,2′-bipyridine (NGENCuB) on activity of pro-MMP9 in MDA-MB-231 breast tumor cells. **A**. Zymography in 1% gelatin-SDS-PAGE. Lane 1: molecular mass marker (M); lane 2: control cells; lanes 3–5: cells treated with NGEN; lane 6: control cells and lanes 7–9: cells treated with NGENCuB (n = 3; 10 µg of total protein was loaded in each lane). A photograph of a representative zymography gel is shown. **B**. pro-MMP9 concentrations were determined by the integrated optical density (IOD) obtained from the bands. Gels were analyzed by densitometry, and activity was expressed as arbitrary units (AU) and data were normalized in percentage compared to untreated control cell lysate. Data represent mean ± SD of three independent assays in triplicate. The results were compared using ANOVA, followed by a Bonferroni's multiple comparison test. Asterisk represents *p≤0.05.

It is known that invasion of basement membranes is mainly mediated by the gelatinase matrix metalloproteinases, MMP2 and 9 [41]. Therefore, we further investigate, through gelatin zymography, whether NGEN and NGENCuB had effects on MMP activity (figure 7AB). For this, MDA-MB-231 cells were seeded and cultured near confluence. Then, monolayers were wounded and incubated in serum-free medium containing NGEN or NGENCuB for 24 hours. After this time, supernatants were collected and tested for MMP activity. We observed that only 100 µM NGENCuB significantly decreased the activity of pro-MMP9 activity, compared to the activity of control cells or NGEN supernatant. Lower (1 and 10 µM) concentrations of NGENCuB had no significant difference on pro-MMP9 activity (Figure 7A, B). Gelatin zymography is particularly useful for the assessment of two key members of MMP family, MMP-2 (gelatinase A) and MMP-9 (gelatinase B), due to their potent gelatin-degrading activity [42], however, since MDA-MB-231 cells do not express MMP-2 [43], [44] we could only demonstrate the effects of NGEN and NGENCuB on pro-MMP9 activity.

**Figure 8:**
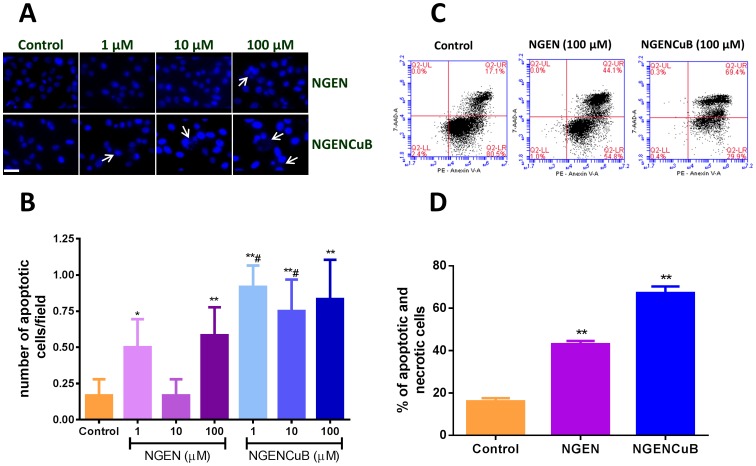
Effects of naringenin (NGEN) or naringenin complexed with copper (II) and 2,2′-bipyridine (NGENCuB) on apoptosis in MDA-MB-231 breast tumor cells. **A**. Nuclear 4', 6-diamidino-2-phenylindole (DAPI) staining. Cells treated or not (control) with different concentrations of NGEN or NGENCuB were observed under a fluorescence microscope. Representative phase-contrast and DAPI staining images were taken 24 h post-treatment. **B**. The number of cells with apoptotic nuclei was counted and plotted in a graphic. Scale bar  = 100 µm. **C**. Cytometry analysis of MDA-MB-231 cells treated or not (control) with 100 µM of NGEN or NGENCuB for 24 hours. After treatment, cells were harvested by trypsinization, centrifuged, washed twice with cold PBS and incubated with PE Annexin V and 7AAD (upper right panel) for 15 minutes in the dark at room temperature and then analyzed by cytometry. **D**. The percentage of apoptotic and necrotic cells was plotted in a graph. Data represent mean ± SD of three independent assays in triplicate. The results were compared using ANOVA, followed by a Tukey's post-hoc analysis. Asterisks represent *p≤0.05, **p≤0.01 (compared to control), #*p*≤0.05 (compared to NGEN).

**Figure 9:**
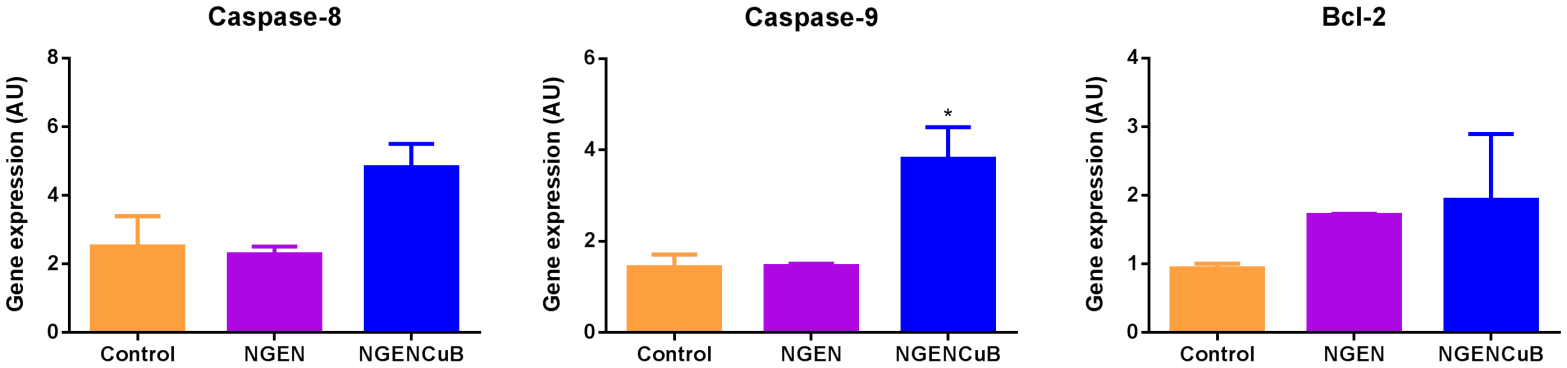
Effects of naringenin (NGEN) or naringenin complexed with copper (II) and 2,2′-bipyridine (NGENCuB) on the expression of apoptotic or anti-apoptotic genes. MDA-MB-231 cells were incubated or not (control) with 1 µM of NGEN or NGENCuB for 24 h. After, total RNA was extracted with Trizol reagent. cDNAs were synthesized and amplification of endogen control (GAPDH) and target genes was run in a Rotor-Gene 6000 real-time rotary analyzer using the gene-specific primer pairs described in MM section. Data represent mean ± SD of three independent assays in triplicate. The results were compared using ANOVA, followed by a Tukey's post-hoc analysis. Asterisk represents *p≤0.05.

We finally accessed the effects of NGEN and NGENCuB on apoptosis using DAPI and Annexin-V staining (figure 8AD). We also tested the effects of NGEN and NGENCuB on gene expression of pro- and anti-apoptotic markers (figure 9). Using DAPI staining we demonstrated that incubation of MDA-MB-231 cells with 100 µM NGENCuB resulted in more apoptotic nuclei, compared with cells incubated with NGEN or control untreated cells (figure 8A, B). Following the same pattern, flow cytometry analysis indicate that 100 µM NGENCuB is significantly more effective inducing apoptosis and necrosis in MDA-MB-231 cells compared with NGEN and control cells (figure 8C, D). NGEN-treated cells generated approximately 40% of apoptotic, whereas NGENCuB produced about 65% of apoptosis in MDA-MB-231 cells (figure 8D). For gene expression analysis we tested two pro-apoptotic genes, caspase-8 and -9 and one anti-apoptotic gene (Bcl-2) and verified that 1 µM NGENCuB, significantly upregulated the expression of caspase-9, compared to NGEN or to untreated control cells. There was no significant difference in the expression of caspase-8 or Blc-2 in tumor cells treated with NGEN or NGENCuB (figure 9). These results indicate the incubation of NGENCuB for 24 hours with MDA-MB-231 cells promotes the activation of intrinsic apoptotic pathway. NGEN itself was not capable of change caspase-8, -9 or even Bcl-2 gene expression (figure 9).

## Discussion

The progression of malignant tumors results from the invasion of the primary tumor to a secondary site, causing metastasis in a multi-step process. These steps can be summarized as follows: cell detachment from the primary tumor, migration into the ECM, intravasation into a blood or lymphatic vessel, survival within the vasculature, adherence of these tumor cells in the endothelium, extravasation, and formation of secondary tumors [45], [46].

Previous studies have showed that naringenin (NGEN) exhibits cytotoxic or inhibitory activity against different cell lines, such as melanoma B16, breast MCF-7, pancreatic aspc-1, and hepatocellular carcinoma tumor cells [47]–[51]. Also, it is well established in the literature that Cu(II) complexes improves anticancer activities in different compounds [52]–[57] mainly because they possess biologically accessible redox potentials and relatively high nucleobase affinity [58]. However, only very few studies have demonstrated the effects of NGEN complexation with metal complexes such as Cu(II) on tumor cells [30], [59], [60]. We did not find any data in the literature reporting the complexation of NGEN with bipyridine. However, it was indicated that bipyridine cisplatin analogues are more effective than cisplatin inducing apoptosis in cancer cell lines [61], [62].

As far as we know, there is only one additional study about the cytotoxic effects of NGEN complexed with Cu(II) on tumor cells [30]. In this work, the authors demonstrated that Cu(II) complexed NGEN showed relatively significant higher inhibitory rate against hepatocellular carcinoma HepG2 cell line than that of NGEN itself. Cytotoxic effects of Cu(II) complexed NGEN against gastric carcinomas from line SGC-7901 and cervical carcinoma HeLa were lower than NGEN. Lee and colleagues [63] studied the effects of NGEN derivatives on colorectal carcinoma RKO cells. Among the derivatives, 7-*O*-benzyl naringenin (KUF-1) and 7-*O*-(m-metoxybenzyl) naringenin (KUF-2) presented IC_50_ of 10 and 15 µM, respectively, whereas NGEN without any modification presented IC_50_ higher than 150 µM. Parker and coworkers analyzed the effects of NGEN on leukemia THP-1 cells and verified an inhibition in cell growth in a concentration-dependent manner, starting from 200 µM [64]. In human leukemia U937 cells, treatment with 50–500 µM NGEN inhibited proliferation in 5%–80% [65]. Finally, in human hepatocellular carcinoma HepG2 cell line, NGEN inhibited cell proliferation in a concentration dependent manner with IC_50_ of 100 µM [66]. In our work, we demonstrated that NGEN inhibited the proliferation of MDA-MB-231 cells with IC_50_ of 342.4 µM, however, the complexation with Cu(II) improved this effect ten times, decreasing the IC_50_ to 32.76 µM. For non-tumor MCF-10A cell line viability, the calculated IC_50_ for NGEN and NGENCuB were 2,833 µM and 907.4 µM, respectively. This could indicate specificity for both compounds, but specially NGENCuB, to affect tumor cells, instead of normal cells' viability. This result could be explained since in fast-dividing tumor cells, the plasma membrane tends to run short of lipids [67], perhaps changing the fluidity of the membrane and facilitating drug penetration.

Other works presented some effects of NGEN complexes, mainly on their antioxidant and DNA binding activities. Naringenin-2-hydroxybenzoylhydrazone (H_5_L), and its Cu(II) complex was found to possess potent antioxidant activity and displayed excellent activity on the superoxide radical [59]. Li and coworkers [60] showed that the complexation of NGEN Shiff-base ligand (H_3_L) with Cu(II) promoted their binding to calf-thymus DNA with more efficacy than that of the ligand (1 µM).

To the best of our knowledge, there are no studies presenting the effects of Cu(II) complexed NGEN on tumor cell migration, MMP activity or apoptosis. Few studies investigated the effects of NGEN, but not Cu(II)-complexed NGEN, in different tumor cell lines. Our work is in agreement with results from Kanno and coworkers [68], who demonstrated that naringenin was able to induce apoptosis in promyeloleukemia HL-60 cell line. Apoptosis was induced by NGEN at concentrations of 100, 250 and 500 µM, via activation of NFkB, caspase-3 and -9 but not caspase-8 pathways. Higher concentration (1 mM) of naringenin caused death via necrosis in this cell line. However, we demonstrate that NGENCuB was more effective generating apoptotic cell death compared to NGEN-treated cells. On the other hand, in our work, in NGEN-treated cells there was no difference in the expression of caspase-9, when compared to control cells. Nevertheless, it is important to notice that we used real time PCR approach to investigate this effect and also that the NGEN concentration used in this assay was very low (1 µM) compared to the work of Kanno and colleagues.

In THP-1 cells NGEN seems to induce mitochondrial damage and apoptosis through modulation of the ratio Bcl-2/Bax, PARP cleavage, activation of caspase-3 and downregulation of Akt pathway [64]. In U937 cells treated with 250 µM of NGEN, morphological analysis with DAPI staining revealed the presence of apoptotic nuclei and flux cytometry analysis confirmed an increased apoptosis ratio in NGEN-treated cells [65]. Incubation of 100 and 200 µM NGEN with HepG2 cells resulted in morphological changes, such as shrink and retraction. Cell staining with DAPI, after treatment with NGEN revealed the appearing of apoptotic bodies. Also, flow cytometry analysis showed that NGEN induced HepG2 cell cycle arrest at the G0/G1 and G2/M phase in a concentration-dependent manner [66].

In summary, the results of this study demonstrate that NGENCuB is more effective than NGEN inhibiting cell proliferation, migration and pro-MMP9 activity, and inducing apoptosis in MDA-MB-231 breast tumor cells. The effect of NGENCuB on apoptosis can be explained, at least in part, by its capacity to upregulate caspase-9 expression.

## Conclusions

Copper (II) and 2,2′-bipyridine complexation improved the chemopreventive effects of naringenin against MDA-MB-231 breast tumor cells. In conclusion, NGEN complexation could be an alternative for the design of a more effective molecule for TN breast cancer treatment and further studies should be performed in order to better understand NGENCuB activities.

## Supporting Information

Figure S1 Infra-Red Spectrum of (±)NGENCuB in KBr.
(TIF)

Figure S2 High Resolution Mass Spectrum of (±)NGENCuB.
(TIF)

Figure S3 Absorption spectrum (10 accumulation) of NGENCuB in DMEN solution for 24 hs. After 24 hours measurements, the absorption spectrum did not change.
(TIF)

Figure S4 TGA curve of NGENCuB.
(TIF)

Figure S5 TGA curve of free NGEN.
(TIF)
