# Supplementary figures and images for: Copper (II) and 2,2′-Bipyridine Complexation Improves Chemopreventive Effects of Naringenin against Breast Tumor Cells
Source: PLoS One. 2014 Sep 5;9(9):e107058. doi: 10.1371/journal.pone.0107058 (PMC4156406; doi:10.1371/journal.pone.0107058)

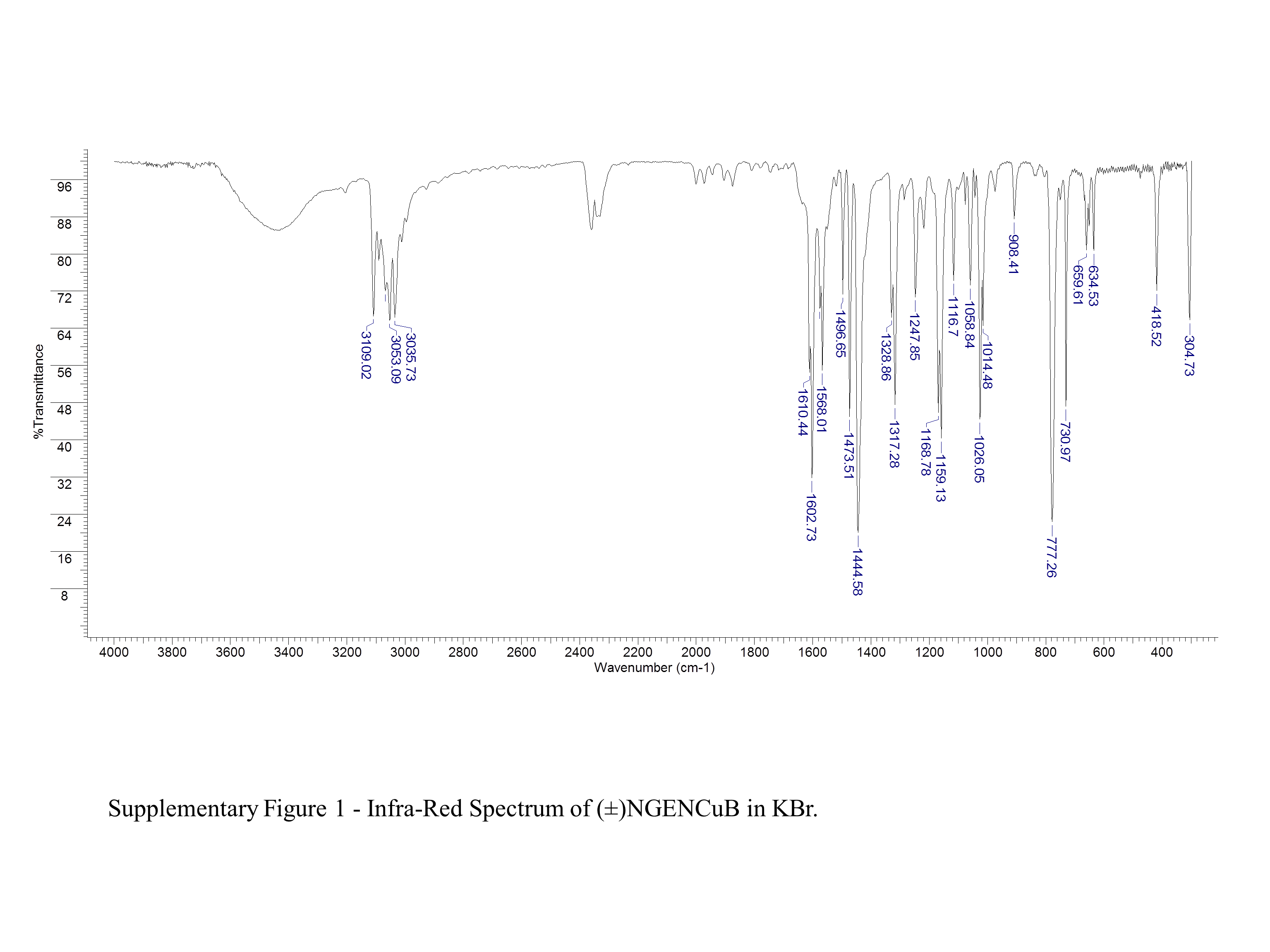

Supplement: Figure S1 — Infra-Red Spectrum of (±)NGENCuB in KBr. (TIF) [file pone.0107058.s001.tif]

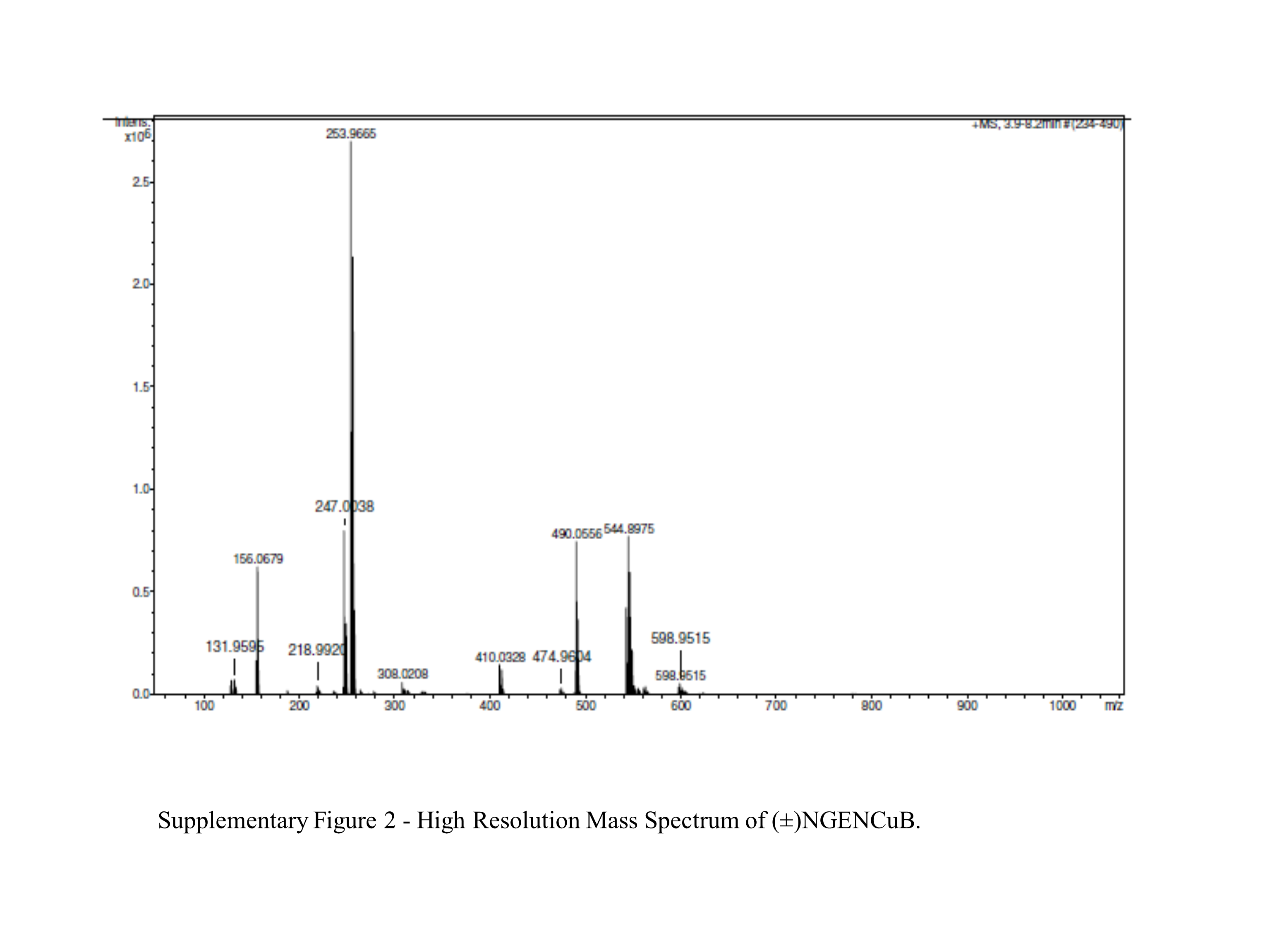

Supplement: Figure S2 — High Resolution Mass Spectrum of (±)NGENCuB. (TIF) [file pone.0107058.s002.tif]

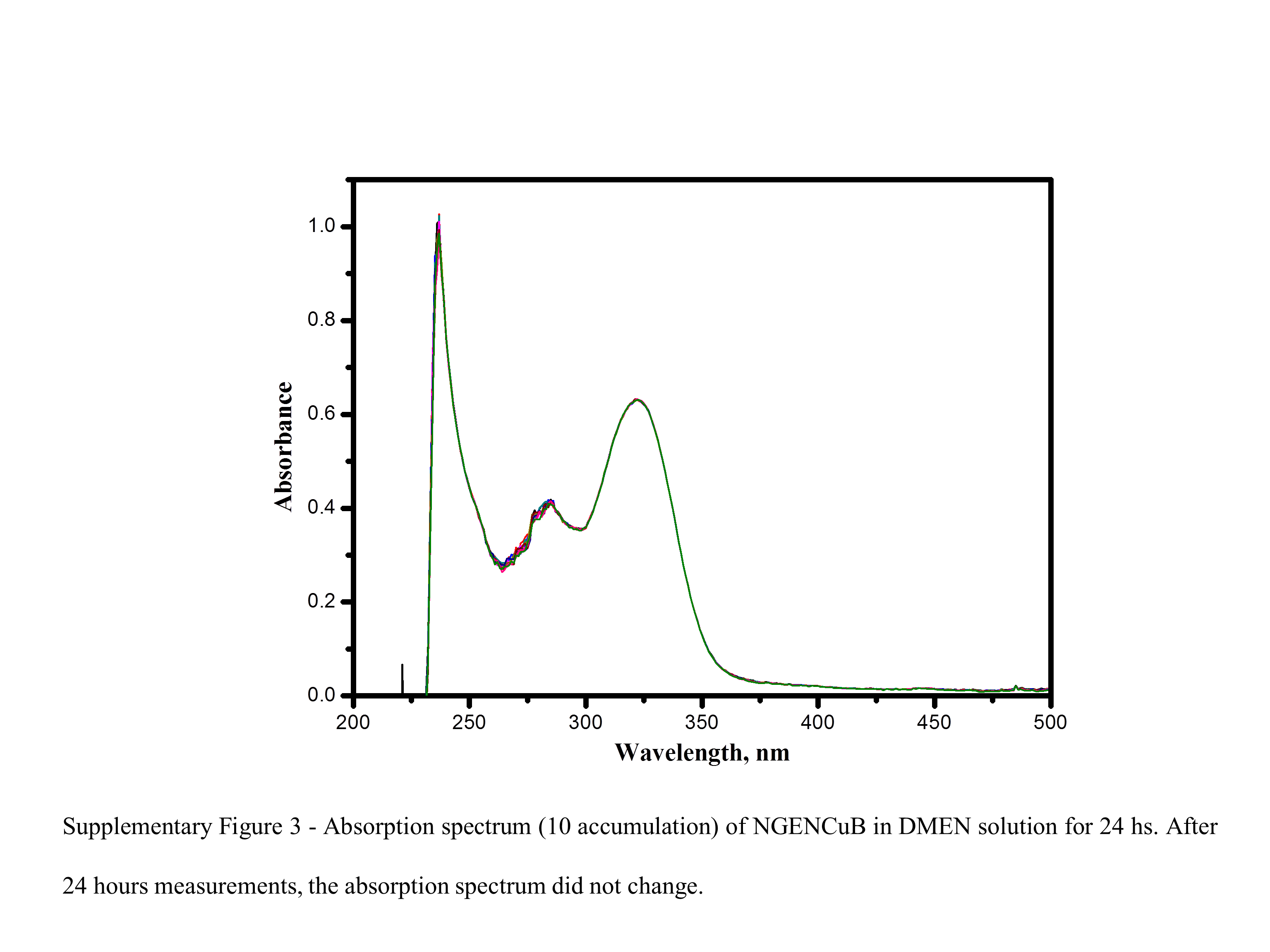

Supplement: Figure S3 — Absorption spectrum (10 accumulation) of NGENCuB in DMEN solution for 24 hs. After 24 hours measurements, the absorption spectrum did not change. (TIF) [file pone.0107058.s003.tif]

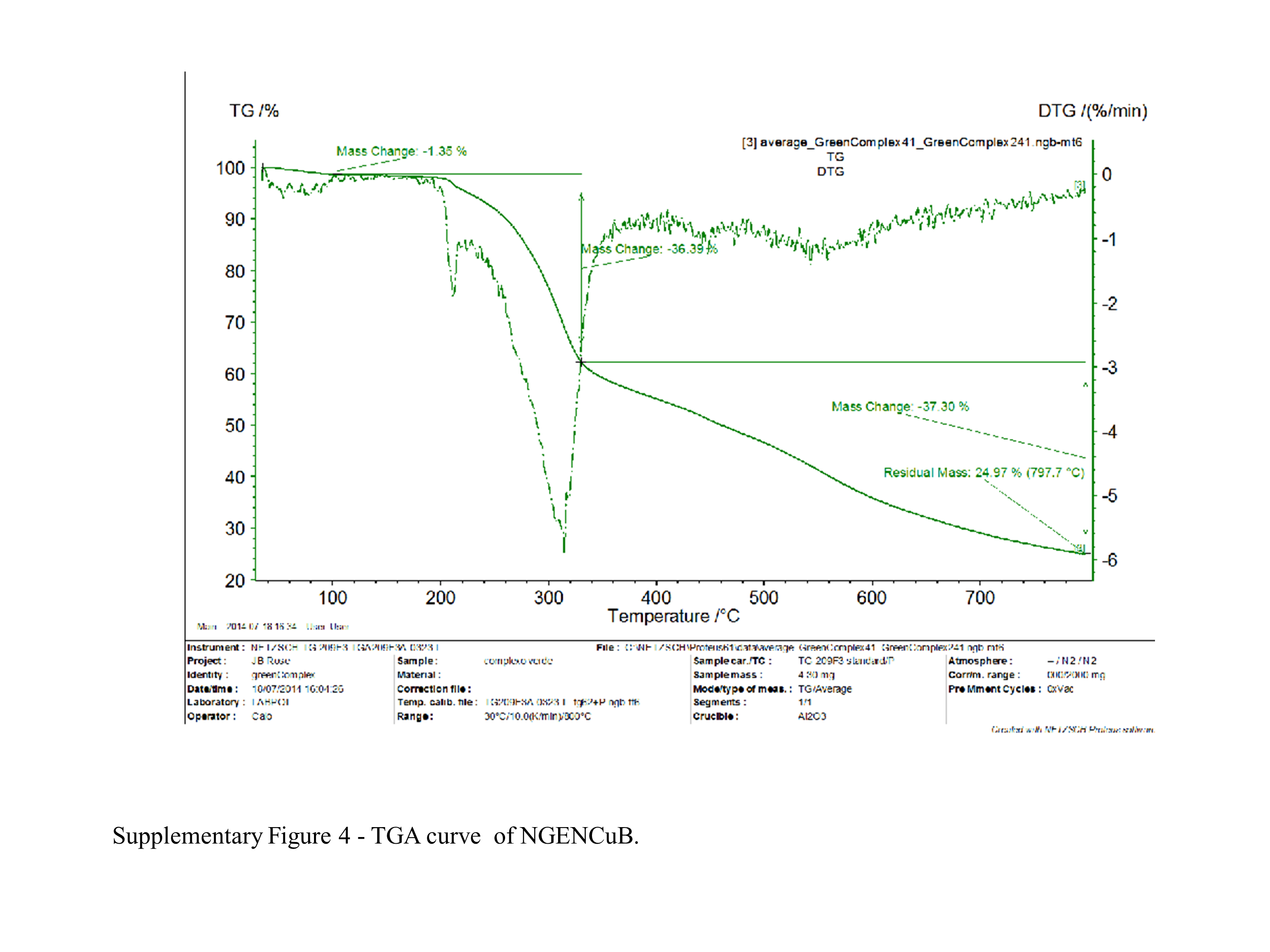

Supplement: Figure S4 — TGA curve of NGENCuB. (TIF) [file pone.0107058.s004.tif]

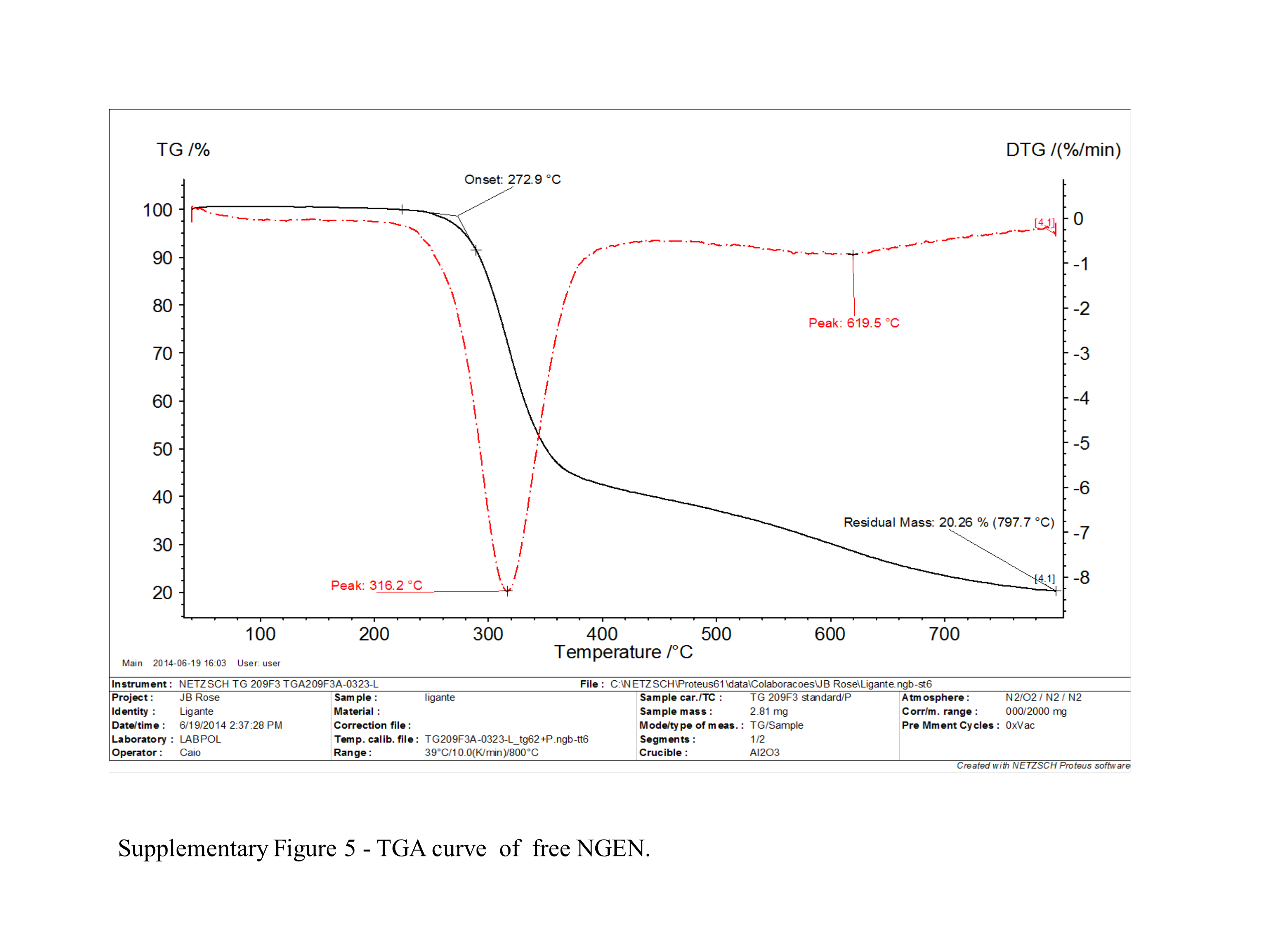

Supplement: Figure S5 — TGA curve of free NGEN. (TIF) [file pone.0107058.s005.tif]
